# Prevalence and Loads of Fecal Pollution Indicators and the Antibiotic Resistance Phenotypes of *Escherichia coli* in Raw Minced Beef in Lebanon

**DOI:** 10.3390/foods9111543

**Published:** 2020-10-26

**Authors:** Issmat I. Kassem, Nivin A Nasser, Joanna Salibi

**Affiliations:** 1 Center for Food Safety, Department of Food Science and Technology, University of Georgia (UGA), Athens, GA 30223-1797, USA; nivin.nasser@uga.edu; 2 Department of Nutrition and Food Sciences, Faculty of Agricultural and Food Sciences, American University of Beirut (AUB), Beirut 1107 2020, Lebanon; jps01@mail.aub.edu

**Keywords:** minced beef meat, antibiotic resistance, *E. coli*, fecal coliforms, food safety, food contamination, Lebanon

## Abstract

Meat is an important source of high biological value proteins as well as many vitamins and minerals. In Lebanon, beef meats, including raw minced beef, are among the most consumed of the meat products. However, minced beef meat can also be an important source of foodborne illnesses. This is of a major concern, because food safety in Lebanon suffers from well-documented challenges. Consequently, the prevalence and loads of fecal coliforms and *Escherichia coli* were quantified to assess the microbiological acceptability of minced beef meat in Lebanon. Additionally, antibiotic resistance phenotypes of the *E. coli* were determined in response to concerns about the emergence of resistance in food matrices in Lebanon. A total of 50 meat samples and 120 *E. coli* isolates were analyzed. Results showed that 98% and 76% of meat samples harbored fecal coliforms and *E. coli* above the microbial acceptance level, respectively. All *E. coli* were resistant to at least one antibiotic, while 35% of the isolates were multidrug-resistant (MDR). The results suggest that Lebanon needs to (1) update food safety systems to track and reduce the levels of potential contamination in important foods and (2) implement programs to control the proliferation of antimicrobial resistance in food systems.

## 1. Introduction

Meat is an integral component of a balanced diet, providing proteins, essential amino acids and various micronutrients such as vitamin B12, niacin, vitamin B6, iron, zinc and phosphorous [1]. Consequently, meat production has increased rapidly over the past 50 years worldwide [2]. Cattle meat production has more than doubled since the 1960s, increasing from 28 million tons in 1961 to 68 million tons in 2014 [2]. In Lebanon, beef meat is among the most consumed meats [3]. The average consumption of beef meat was 39.63 kg/capita in Lebanon [2] and the mean intake was 47.6 g/day in surveyed consumers in Beirut, the capital of Lebanon [3]. Lebanon produced 47,484 tons of beef meat in 2014 [2]; however, beef meat in the Lebanese market is mainly imported from Brazil and India [4]. Beef meat constitutes a popular and economically important food in Lebanon. Notably, beef meat can also be consumed raw as part of famous Lebanese dishes. However, Lebanon has been witnessing challenges in food safety that have received media attention and raised national public health concerns. This was not surprising, because food safety can severely impact the health of a population [5], especially in developing countries with weakened infrastructure and limited resources [6]. Foodborne illnesses have been estimated to affect almost 1 in 10 people (~600 million individuals), while 420,000 die every year [5]. Notably, children less than 5 years old constitute a third of the deaths associated with foodborne diseases [5]. In developing countries, the detrimental manifestations of foodborne disease are highly expressed, claiming or debilitating the lives of the most vulnerable populations such as the children, the elderly, the nutritionally deprived, and the immunocompromised [7]. Foodborne diseases in these countries perpetuate the cycle of poverty and damage budding national brands and already-fragile economies. Taken together, this necessitated a closer examination of the safety of popular and essential foods, including minced beef meat, in Lebanon.

Beef meat can be contaminated by different foodborne pathogens that can cause severe illnesses in consumers and have been implicated in foodborne outbreaks [8]. Studies have detected *Escherichia coli* (*E. coli*), including *E. coli* O157:H7, *Salmonella* spp., *Listeria monocytogenes* and *Campylobacter* spp. in minced beef. Minced beef can be contaminated in a variety of ways. For example, during mincing, bacteria that contaminate the surface of the meat/carcass can be mixed into the minced product. Adding leftover cuts and trims during mincing can also increase bacterial loads in comparison to the original fresh carcass or cut. Lack of hygienic practices during processing and the cleanliness of the equipment can contribute to the contamination of the meat [9]. Furthermore, mincing might cause a slight increase in the temperature of the product allowing bacteria to grow faster [9]. Being a nutrient-rich matrix, minced beef becomes a favored environment for the growth of various microorganisms that can be harmful to consumers [10]. Therefore, the microbial quality of minced beef should be closely monitored, especially when the meat will be consumed undercooked or raw, which heightens the risks of infections in consumers.

Foodborne infections are becoming increasingly problematic because of the rise of foodborne bacterial pathogens that have acquired resistance to antibiotics. This can lead to hard-to-treat and complicated infections in consumers [11]. The occurrence of antibiotic resistant bacterial pathogens in various foods, including poultry- and beef products is well documented [12,13,14,15]. In fact, food and food production have been implicated in the emergence and spread of antimicrobial resistance (AMR); largely due to the misuse and/or overuse of clinically important antibiotics in food-animal farming practices [16,17,18,19]. Antibiotics have been used heavily in food-animals to treat (therapy) or prevent (prophylaxis) diseases and for growth promotion. While this has received considerable scrutiny, the use of antibiotics in growth promotion has been the most controversial, because of its potential role in facilitating the emergence of AMR in food animal operations [16]. Currently, the World Health Organization (WHO) recognizes AMR as an imminent crisis, which is predicted to have a devastating impact on health and economy across the globe [16,17,18,19]. However, AMR bacteria in Lebanon in food and environmental matrices have received little attention [17,20]. Therefore, it is important to investigate AMR in minced beef and other foods in Lebanon.

In developing countries, such as Lebanon, it is difficult to estimate the burden of foodborne diseases because of inconsistent and weak monitoring and surveillance systems. The country is also facing a severe economic crisis that has limited these systems further. Although Lebanon has a modern Food Safety Law (issued in 2016), it has not been implemented because of weak governmental oversight. Currently, there are Lebanese standard specifications of minced beef meat (standard 503:2004) that were set by the Lebanese Standards Institution (LIBNOR). However, the standard is outdated (issued in 2004) and should reflect current data collected in the country [21,22]. Admittedly, original data on microbiological criteria that affect the acceptability of many foods are scant in Lebanon. Therefore, we launched an investigation to monitor potential microbial contaminations of different foods in Lebanon. Here, we focused on the prevalence and loads of fecal indicators, fecal coliforms and *E. coli,* in raw minced beef in the Lebanese market. Indicator organisms are widely used to assess the microbiological criteria of foods and the detection of fecal indicators, such as *E. coli* and fecal coliforms, suggests the presence of enteric pathogens in foods [23]. Furthermore, acquired resistance in *E. coli* can reflect the use of antibiotics because of the selection pressure that is needed for the persistence of AMR strains [24]. Indeed, *E. coli* has been used as an indicator for monitoring antibiotic resistance in foods [24]. Therefore, we also analyzed the AMR profiles of the *E. coli* associated with minced beef meat.

## 2. Materials and Methods

### 2.1. Sampling of Minced Beef

During the spring and summer of 2018, a total of 50 samples of raw minced beef were collected from 50 different butcheries and grocery stores in Beirut, Lebanon. The samples were immediately placed on ice, transported to the laboratory, and processed within 2–3 h of collection.

### 2.2. Enumeration of Fecal Coliforms and E. coli

For each sample, minced beef meat was weighed, and 25 g were aseptically placed in a sterile stomacher bag (Fisher Scientific, New Hampshire, USA). The sample was then diluted 1:10 with 225 mL of sterile buffered peptone water (BPW) (Oxoid, Hampshire, UK) and homogenized for 1 min using a stomacher (Thomas Scientific, New Jersey, USA). The suspension was then serially diluted (10-fold) in BPW and 3 dilutions (10^−1^, 10^−2^, 10^−4^) were plated on RAPID’*E. coli* 2 agar plates (BioRad, Hercules, California, USA) in duplicates. The plates were incubated at 44 °C for 18–24 h under aerobic conditions. Colony forming units (CFU) that matched the diagnostic phenotypes (violet to pink *E. coli* colonies and blue to green colonies for other fecal coliforms) were counted and bacteria densities were determined by averaging the counts from the duplicates. Data were reported as averages of CFU per gram of raw minced beef. BPW without meat was used as control throughout the experiment. *E. coli* DH5α was used to test the quality of the RAPID’*E. coli* 2 agar plates. Average CFU counts of *E. coli* and fecal coliforms were compared using the Student’s *t*-test. A *p* < 0.05 was used to identify statistically significant differences.

### 2.3. Assessing Antimicrobial Resistance (AMR) of E. coli Using the Disk Diffusion Assay

Antimicrobial resistance analysis was performed on *E. coli* isolated from the minced beef samples using the disk diffusion assay [25]. Briefly, 120 *E. coli* were isolated from contaminated meat samples (1 to 3 isolates per sample). *E. coli* isolates were suspended in Mueller-Hinton (MH) broth (Oxoid, Hampshire, UK) and their turbidity was adjusted using a 0.5 McFarland standard and a spectrophotometer (Thermo Fisher Scientific, Massachusetts, USA) [25]. The bacterial suspensions were then spread on MH agar (MH) plates. Nineteen different antibiotic discs (Oxoid, Hampshire, UK) belonging to 9 classes of antibiotics were then added on top of the MH agar plates. The antibiotic classes and discs used were: (1) penicillins (class): penicillin (PEN; 6 µg) and ampicillin (AMP; 10 μg); (2) β-lactam/β-lactamase inhibitor combinations: amoxicillin/clavulanic acid (AMC; 20 μg/10 μg); (3) cephalosporins: cefepime (FEP; 30 μg), cefotaxime (CTX; 30 μg), cefalexin (LEX; 30 μg), and cefixime (CFM; 6 μg); (4) carbapenems: doripenem (DOR; 10 μg), meropenem (MEM; 10 μg), and imipenem (IPM; 10 μg); (5) aminoglycosides: gentamicin (GEN; 10 μg), kanamycin (KAN; 30 μg), and streptomycin (STR; 10 μg); (6) tetracyclines: tetracycline (TET; 30 μg); (7) quinolones and fluoroquinolones: ciprofloxacin (CIP; 5 μg) and norfloxacin (NOR; 10 μg); (8) folate-pathway inhibitors: trimethoprim/sulfamethoxazole (SXT; 25 μg); and (9) phenicols: chloramphenicol (CHL; 30 μg) [26]. These antibiotics are (1) clinically and agriculturally important and (2) used to evaluate acquired resistance in *Enterobacteriaceae*, including *E. coli* [27,28]. Erythromycin (ERY; 15 μg) was used for quality control, because *E. coli* is intrinsically resistant to this antibiotic [29]. Additionally, *E. coli* DH5α was also used for quality control across the experiments. The MH agar plates were incubated at 37 °C for 18–24 h. The zone of inhibition was measured and the AMR profiles of the isolates were determined using the Clinical and Laboratory Standards Institute (CLSI) and the European Committee on Antimicrobial Susceptibility Testing (EUCAST) standards [27,28]. The AMR phenotypes were analyzed using hierarchical clustering. Briefly, the AMR profile of each strain was coded in Excel^®^ (Microsoft, Washington, USA) as follows: 1 corresponded to resistance, while 0 and −1 replaced intermediate resistance and susceptibility, respectively. This resulted in a matrix comprised of AMR profiles of the strains in rows, while the tested antibiotics were represented in the column. Following this, the matrix was exported to MeV v4.6.2 software (http://www.tm4.org/) [30] to perform hierarchical clustering using the Pearson correlation as a distance metric and the complete linkage method [31]. A graphical presentation of the matrix (heat map) was produced, where the upper limit (1) was colored green, while midpoint (0) and lowest limit (−1) were colored black and red, respectively [31].

## 3. Results and Discussion

### 3.1. Prevalence and Loads of Fecal Coliforms and E. coli

**Figure 1:**
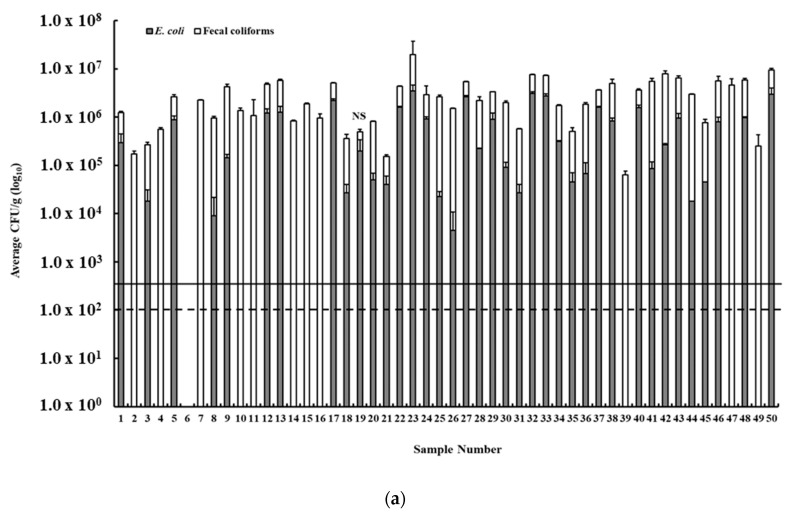

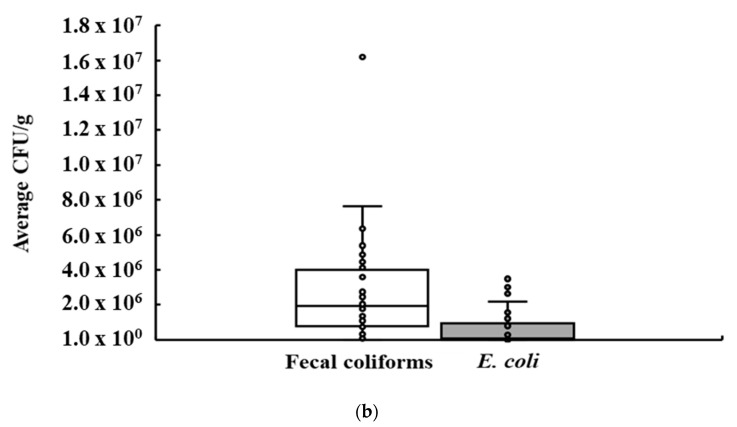
(**a**) The prevalence and loads (colony forming unit (CFU)/g) of fecal coliforms and *E. coli* in minced beef in Lebanon. The dashed lines show the limit of acceptability based on the Lebanese Standards Institution (LIBNOR) (fecal coliforms; 100 CFU/g), while the black line indicates *E. coli* limit adopted in other countries (500 CFU/g). NS: indicates no significant difference between the fecal coliforms and *E. coli* CFU. (**b**) Box and whisker plot showing the variation in the average CFU/g (circles) of *E. coli* and fecal coliforms in the minced beef samples.

We assessed the acceptability of the minced beef samples in Lebanon by quantifying the prevalence and loads of fecal indicators, namely fecal coliforms and *E. coli*. Fecal coliforms were present in 49 of 50 (98%) raw minced beef samples, while *E. coli* was detected in 38 samples (76%). Fecal coliform CFU counts in positive samples ranged between 6.3 × 10^4^ CFU/g and 1.62 × 10^7^ CFU/g, while *E. coli* CFU counts ranged between 4.5 × 10^3^ CFU/g and 3.48 × 10^6^ CFU/g (Figure 1a). The visual distribution of the CFU counts of fecal coliforms in comparison to those of *E. coli* is presented in Figure 1b, which also shows the median, quartiles (25% and 75%), minimum and maximum values, and outliers.

According to LIBNOR standards, the acceptable limit of fecal coliforms in minced beef is 100 CFU/g [21], which is similar to other countries such as New Zealand that has a limit ranging from 100 to 1000 CFU/g [32], while the USA uses 1000 CFU/g of total coliforms as a critical limit for ground beef [32]. Therefore, using the LIBNOR standards and those from other countries, 98% of the tested minced beef samples in Lebanon exceeded the microbiological criterion based on fecal coliform counts (Figure 1a). The LIBNOR standard [21] for minced raw meat does not include limits on *E. coli* counts. However, there are many countries and researchers who established an *E. coli* limit to determine the acceptability of meat. For example, the USA uses 500 *E. coli* CFU/g as a critical limit for boneless beef [33]. Other studies showed that some states have more strict limits. For example, Oregon State (USA) has set a maximum of 50 CFU/g of *E. coli* [34], while New York State (USA) adopted 10 CFU/g of *E. coli* for assessing the acceptability of minced meat products [34]. Furthermore, the European Union (EU) Commission Regulation (EC) No. 2073/2005 set the limit of *E. coli* at 50 CFU/g [35]. When considering the aforementioned limits, it can be concluded that 76% of the tested minced beef samples in Lebanon exceeded the microbiological criterion based on *E. coli* counts.

Taken together, the wide prevalence and high loads of fecal indicator bacteria on minced beef samples in Lebanon are concerning, perhaps suggesting the need for better hygienic practices in the minced beef production chain. Admittedly, assessing the hygienic practices and possible sources of contamination of raw minced beef in a sustainable manner can be challenging. Specifically, monitoring must encompass (1) different food iterations that may contain raw beef in Lebanon (a notable example is minced beef with mixed herbs, Middle Eastern spices and onions that are consumed raw or cooked), (2) different types of indicator organisms and pathogens that might be present in each product, (3) differences in the handling and/ or processing procedures in foodservice establishments, butcher shops, and meat processing plants, and (4) a variety of sampling and testing procedures that require specialized laboratories, equipment and trained personnel [36,37]; all of which are difficult to secure in a country that is experiencing severe political and economic crises. However, despite the challenges, the safety of meat as well as other essential foods is critical to maintain public health and should be prioritized by the Lebanese government.

Both fecal coliforms and *E. coli* are effective indicators of fecal pollution. The latter suggests the presence of a variety of pathogens that can negatively impact human health [23,38]. In this study, 98% and 76% of the meat samples were rejected based on counts of fecal coliforms and *E. coli*, respectively, showing a difference in the rate of rejection based on the tested indicator. Subsequently, Lebanon would benefit from studies that screen for the best indicators of meat acceptability. This will reduce monitoring costs and will perhaps better reflect the acceptability of the meat. LIBNOR, along with other specialized stakeholders, should work on modifying and updating the current safety guidelines to enable proper assessment of the microbial quality of raw meat products in Lebanon.

### 3.2. AMR Profiles of E. coli Isolated from Minced Beef Meat

**Figure 2:**
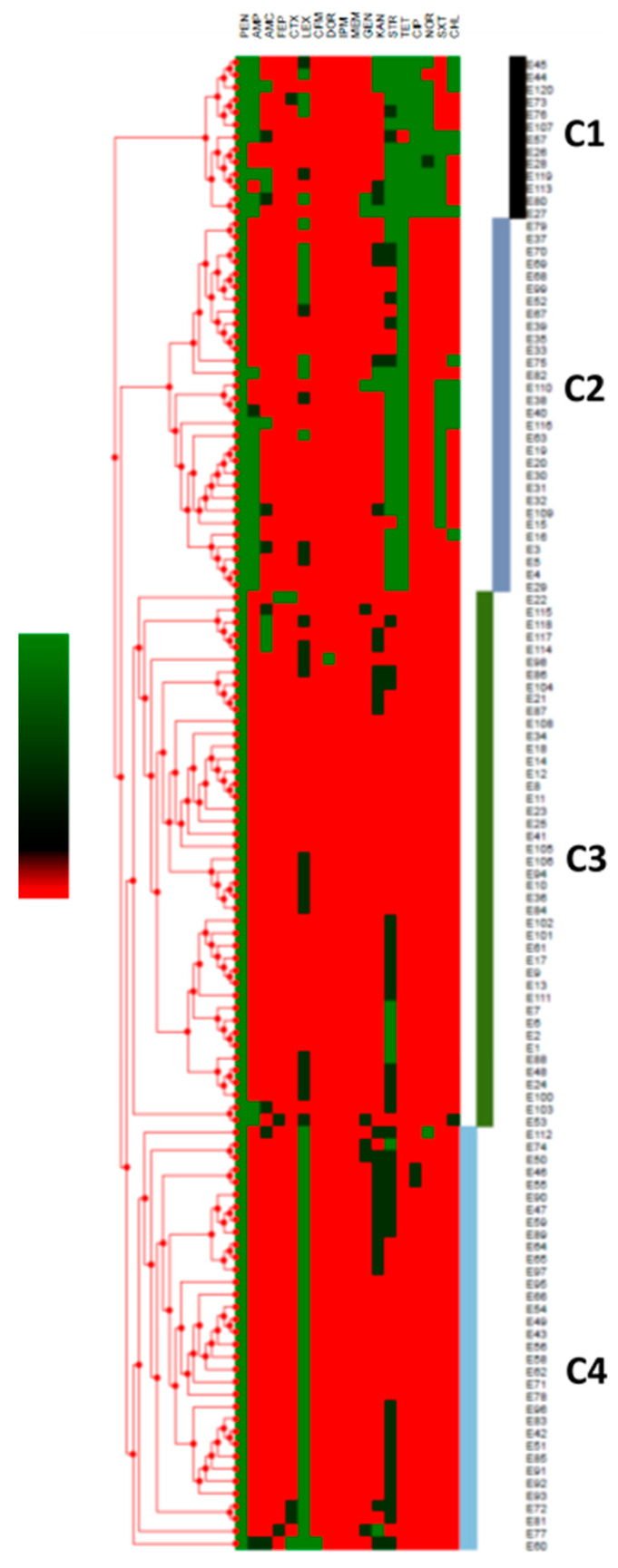
Hierarchical clustering of the antimicrobial resistance (AMR) profiles of each *E. coli* (*n* = 120) isolated from minced beef meat in Lebanon. The strains were labelled E1 to E120. The green color in the scale and the heat map represents resistance, while black and red indicate intermediate resistance and susceptibility, respectively. The letter C indicates clusters.

AMR analysis revealed that the *E. coli* isolates (*n* = 120) were resistant to PEN (100% of isolates), AMP (22.5%), FEP (0.8%), CTX (1.7%), LEX (37.5%), DOR (0.8%), GEN (2.5%), KAN (5.8%), STR (30%), TET (34.2%), CIP (10.8%), NOR (10%), SXT (15.8%), and CHL (10%) (Table 1). All isolates were sensitive to AMC, CFM, IPM, and MEM. Furthermore, 35% of the *E. coli* were classified as multi-drug resistant (resistance to ≥three classes of antibiotics) [39] (Table 1). Notably, three and 12 isolates exhibited resistance to six and five classes of antibiotics, respectively (Table 1). Hierarchal clustering organized the AMR profiles into four distinct clusters (Figure 2). Resistance to CIP and NOR was notable in the AMR profiles of isolates in Cluster 1 (C1). Similarly, resistance to STR–TET and resistance to LEX were notable in C2 and C4, respectively (Figure 2). The majority of multi-drug resistant (MDR) *E. coli* were grouped in C1 and C2, while C3 and C4 were mainly comprised of *E. coli* that were resistant to one or two antibiotics (Figure 2).

In comparison, 55% of *E. coli* isolated from raw beef preparations in Northwest Spain were resistant to TET [40]. Additionally, 6.67% of the *E. coli* isolated from beef muscles in Ghana were resistant to AMC, CHL, and GEN, respectively [41]. In the same study, 40% and 0% of the isolates were resistant to TET and CIP, respectively [41]. In Egypt, 25% of the *E. coli* isolated from raw samples were resistant to TET, STR, and AMP, respectively [42]. This suggested that the resistance in Lebanon was comparable to those in other countries. However, it should be noted that beef growing operations are limited in Lebanon in comparison to the aforementioned countries. Furthermore, the size of Lebanon and its human population are much smaller in comparison to these countries. Taken together, this suggested that (1) there is an overreliance on the use of antibiotics in Lebanese farming [17,43,44] and/or (2) the resistance percentages are masked by the beef imported from other countries. Regardless, given that *E. coli* is an indicator for monitoring antibiotic resistance in foods, the AMR detected in Lebanese raw minced beef samples is a cause of concern. Therefore, the prevalence of multi-drug resistant *E. coli* isolates in the raw beef meat samples in Lebanon should be monitored periodically to assess the dissemination of these bacteria and the potential proliferation of transmissible genetic determinants of resistance [43,44,45]. Given the potential negative impacts on consumers, environment, and farmed animals, decreasing the incidences of multi-drug resistance in cattle and particularly in beef is a necessity.

Many studies have reported a high number of MDR pathogens associated with foods, food-animals and food-environment, including *E. coli*, *Campylobacter* spp., and *Salmonella* spp. [42,43,44,45,46,47]. The emergence and proliferation of resistance have been linked to the improper use of antibiotics in medical and agricultural practices [16,17,48,49]. In the latter, AMR bacteria and genes encoding resistance can be transmitted to humans via (1) the food chain, (2) direct farm exposure, and/or (3) environmental contamination with farm waste and products [48,49,50]. As a result, recalcitrant AMR infections associated with food and/or agriculture have become a global risk [11,16,17,18,19]. This is predicted to be more severe in developing countries that have challenges in infrastructure and antimicrobial stewardship [17,18,19]. Lebanon is one of these countries that have also been affected by wide-spread pollution. Therefore, the emergence and dissemination of resistance in Lebanon should receive national and global attention, because AMR bacteria and genes can spread locally and beyond national borders, affecting other countries [43,51].

## 4. Conclusions

This study revealed a high occurrence of fecal coliforms and *E. coli* in raw minced beef in the Lebanese market. The high loads of these indicator bacteria are worrying, especially because beef meat is a major food item in the Lebanese cuisine and is consumed raw or minimally cooked in many instances. The occurrence of multi-drug resistant *E. coli* in the meat highlights the importance of adopting and implementing policies for reducing the use of antibiotics in food animal production in Lebanon and countries of importation. Furthermore, it might be beneficial to investigate and monitor the genetic bases of AMR in *E. coli* isolated from minced beef in order to assess the possibility of the persistence and dissemination of resistance to other vital matrices in Lebanon. Here, we call for updating national food safety systems in Lebanon and for investing in infrastructure and expertise that allow sustainable monitoring of food safety and the spread of AMR. This will greatly benefit public health and the economy in Lebanon.

## Figures and Tables

**Table 1:** Antibiotic resistance profile of *E. coli* isolated from raw minced meat in Lebanon. Antibiotics in the resistance profile were arranged according to the order of antibiotics/classes listed in the Clinical and Laboratory Standards Institute (CLSI) guidelines. Penicillin (PEN), ampicillin (AMP), cefepime (FEP), cefotaxime (CTX), cephalexin (LEX), doripenem (DOR), gentamicin (GEN), kanamycin (KAN), streptomycin (STR), tetracycline (TET), ciprofloxacin (CIP), norfloxacin (NOR), trimethoprim-sulfamethoxazole (SXT), and chloramphenicol (CHL). All isolates were susceptible to amoxicillin/clavulanic acid (AMC), cefixime (CFM), meropenem (MEM), and imipenem (IPM), so these antibiotics were not listed in the table. Shaded (grey) cells with the letter R indicate resistance.

Resistance Profiles	PEN	AMP	FEP	CTX	LEX	DOR	GEN	KAN	STR	TET	CIP	NOR	SXT	CHL	Number of Isolates (%)	Number of Antibiotic Classes
P1	R	R					R	R	R	R	R	R	R	R	1 (0.8)	6
P2	R	R			R		R		R	R	R	R	R		1 (0.8)	6
P3	R	R			R			R	R	R	R			R	1 (0.8)	6
P4	R	R						R	R	R	R	R		R	2 (1.7)	5
P5	R	R							R	R	R	R	R		1 (0.8)	5
P6	R	R			R				R	R	R	R			2 (1.7)	5
P7	R						R	R	R	R			R	R	1 (0.8)	5
P8	R	R							R	R			R	R	1 (0.8)	5
P9	R	R									R	R	R	R	1 (0.8)	4
P10	R	R			R			R	R	R					1 (0.8)	4
P11	R									R	R	R	R	R	1 (0.8)	5
P12	R								R	R	R	R	R		1 (0.8)	5
P13	R	R							R	R				R	1 (0.8)	4
P14	R	R							R		R	R			1 (0.8)	3
P15	R	R							R	R			R		7 (5.8)	4
P16	R								R	R			R	R	2 (1.7)	5
P17	R								R	R	R		R		1 (0.8)	5
P18	R	R							R	R					4 (3.3)	3
P19	R	R								R			R		1 (0.8)	3
P20	R				R					R				R	1 (0.8)	4
P21	R				R				R	R					1 (0.8)	4
P22	R				R			R							1 (0.8)	3
P23	R				R							R			1 (0.8)	3
P24	R				R				R						1 (0.8)	3
P25	R				R					R					5 (4.2)	3
P26	R								R	R					1 (0.8)	3
P27	R			R	R										1 (0.8)	2
P28	R		R	R											1 (0.8)	2
P29	R	R													2 (1.7)	1
P30	R				R										29 (24.2)	2
P31	R								R						5 (4.2)	2
P32	R									R					4 (3.3)	2
P33	R					R									1 (0.8)	2
P34	R														35 (29.2)	1
Total															120	

## References

[1] Williams P.G. (2007). Nutritional Composition of Red Meat. https://ro.uow.edu.au/hbspapers/48.

[2] Ritchie H., Roser M. (2017). Meat and Seafood Production & Consumption. Our World in Data. https://ourworldindata.org/grapher/meat-production-tonnes.

[3] Nasreddine L., Hwalla N., Sibai A., Hamzé M., Parent-Massin D. (2006). Food consumption patterns in an adult urban population in Beirut, Lebanon. Public Health Nutr..

[4] USDA FAS (2016). Global Agricultural Information Network (GAIN): Lebanese Market Overview. https://gain.fas.usda.gov/Recent%20GAIN%20Publications/Lebanese%20Market%20Overview_Cairo_Lebanon_6-26-2016.pdf.

[5] WHO (2017). Food Safety. http://www.who.int/news-room/fact-sheets/detail/food-safety.

[6] UNHCR UNHCR Lebanon-Operational Fact Sheet-January 2020. https://www.unhcr.org/lb/wp-content/uploads/sites/16/2020/02/UNHCR-Lebanon-Operational-Fact-sheet-January-2020.pdf.

[7] Todd E. (2014). Foodborne diseases: Overview of biological hazards and foodborne diseases. Encycl. Food Saf..

[8] Jay J.M., Loessner M.J., Golden D.A. (2005). Foodborne gastroenteritis. Modern Food Microbiology.

[9] FSIS (1999). FSIS Microbiological Hazard Identification Guide for Meat and Poultry Components of Products Produced by very Small Plants. https://www.fsis.usda.gov/wps/wcm/connect/0b96c2cf-ffdc-4dc5-92f0-bdfd181b8368/higuide.pdf?MOD=AJPERES.

[10] NSAI (2007). IS 340:2007 Hygiene in the Catering Sector.

[11] Economou V., Gousia P. (2015). Agriculture and food animals as a source of antimicrobial-resistant bacteria. Infect. Drug Resist..

[12] Johnson J.R., Sannes M.R., Croy C., Johnston B., Clabots C., Kuskowski M.A., Bender J., Smith K.E., Winokur P.L., Belongia E.A. (2007). Antimicrobial drug–resistant *Escherichia coli* from humans and poultry products, Minnesota and Wisconsin, 2002–2004. Emerg. Infect. Dis..

[13] Moawad A.A., Hotzel H., Awad O., Tomaso H., Neubauer H., Hafez H.M., El-Adawy H. (2017). Occurrence of *Salmonella enterica* and *Escherichia coli* in raw chicken and beef meat in northern Egypt and dissemination of their antibiotic resistance markers. Gut. Pathog..

[14] Nekouei O., Checkley S., Waldner C., Smith B.A., Invik J., Carson C., Avery B., Sanchez J., Gow S. (2018). Exposure to antimicrobial-resistant *Escherichia coli* through the consumption of ground beef in Western Canada. Int. J. Food Microbiol..

[15] Rasheed M.U., Thajuddin N., Ahamed P., Teklemariam Z., Jamil K. (2014). Antimicrobial drug resistance in strains of *Escherichia coli* isolated from food sources. Rev. Inst. Med. Trop. Sao Paulo.

[16] Kassem I.I., Helmy Y.A., Kashoma I.P., Rajashekara G., Ricke S. (2016). The Emergence of Antibiotic Resistance in Poultry Farms. Achieving Sustainable Production of Poultry Meat Volume 1: Safety, Quality and Sustainability.

[17] Kassem I.I., Hijazi M.A., Saab R. (2019). On a collision course: The availability and use of colistin-containing drugs in human therapeutics and food-animal farming in Lebanon. J. Glob. Antimicrob. Resist..

[18] WHO (2020). Antimicrobial Resistance. https://www.who.int/news-room/fact-sheets/detail/antimicrobial-resistance.

[19] WHO (2017). Antimicrobial Resistance in the Food Chain. https://www.who.int/foodsafety/areas_work/antimicrobial-resistance/amrfoodchain/en/.

[20] Osman M., Al Mir H., Rafei R., Dabboussi F., Madec J.-Y., Haenni M., Hamze M. (2019). Epidemiology of antimicrobial resistance in Lebanese extra-hospital settings: An overview. J. Glob. Antimicrob. Resist..

[21] LIBNOR (2004). Lebanese Specification Standard No. 503: Minced Meat.

[22] El-Jardali F., Hammoud R., Kamleh R., Jurdi M. (2014). K2P Briefing Note: Protecting Consumers in Lebanon: The Need for Effective Food Safety System.

[23] Erkmen O., Bozoglu T.F. (2016). Indicators of Foodborne Pathogens.

[24] EFSA (2008). Report from the Task Force on Zoonoses Data Collection including guidance for harmonized monitoring and reporting of antimicrobial resistance in commensal *Escherichia coli* and *Enterococcus* spp. from food animals. EFSA J..

[25] Balouiri M., Sadiki M., Ibnsouda S.K. (2016). Methods for in vitro evaluating antimicrobial activity: A review. J. Pharm. Anal..

[26] Sourenian T., Mann D., Li S., Deng X., Jaafar H., Kassem I.I. (2020). Dissemination of multidrug-resistant *Escherichia coli* harboring the mobile colistin resistance gene *mcr-1.1* on transmissible plasmids in the Mediterranean Sea. J. Glob. Antimicrob. Resist..

[27] Nguyen M.C.P., Woerther P.L., Bouvet M., Andremont A., Leclercq R., Canu A. (2009). *Escherichia coli* as reservoir for macrolide resistance genes. Emerg. Infect. Dis..

[28] CLSI (2016). Performance Standards for Antimicrobial Susceptibility Testing.

[29] European Committee on Antimicrobial Susceptibility Testing (EUCAST) (2018). Breakpoint Tables for Interpretation of MICs and Zone Diameters; EUCAST, Version 8.1. http://www.eucast.org.

[30] Multiple Experiment Viewer (MEV). http://mev.tm4.org/#/welcome.

[31] Berrazeg M., Drissi M., Medjahed L., Rolain J.M. (2013). Hierarchical clustering as a rapid tool for surveillance of emerging antibiotic-resistance phenotypes in *Klebsiella pneumoniae* strains. J. Med. Microbiol..

[32] Government of New Zealand (1995). Microbiological Reference Criteria for Food. https://www.mpi.govt.nz/dmsdocument/21185/direct.

[33] AMS USDA (2020). Microbiological Testing of Ams Purchased Meat, Poultry and Egg Commodities. https://www.ams.usda.gov/resources/microbiological-testing.

[34] Goepfert J. (1976). The aerobic plate count, coliform and *Escherichia coli* content of raw ground beef at the retail level. J. Milk Food Technol..

[35] EC (2005). Commission Regulation (EC) No 2073/2005 of 15 November 2005 on microbiological criteria for foodstuffs. OJEU.

[36] National Research Council (1985). Application of microbiological criteria to foods and food ingredients. An Evaluation of the Role of Microbiological Criteria for Foods and Food Ingredients.

[37] National Research Council (1985). Current status of microbiological criteria and legislative bases. An Evaluation of the Role of Microbiological Criteria for Foods and Food Ingredients.

[38] Ekici G., Dümen E., Starčič Erjavec M. (2019). Escherichia coli and food safety. The Universe of Escherichia coli.

[39] Souli M., Galani I., Giamarellou H. (2008). Emergence of extensively drug-resistant and pandrug-resistant Gram-negative bacilli in Europe. Eurosurveillance.

[40] González-Gutiérrez M., García-Fernández C., Alonso-Calleja C., Capita R. (2020). Microbial load and antibiotic resistance in raw beef preparations from northwest Spain. Food Sci. Nutr..

[41] Adzitey F. (2020). Incidence and antimicrobial susceptibility of *Escherichia coli* isolated from beef (meat muscle, liver and kidney) samples in Wa Abattoir, Ghana. Cogent Food Agric..

[42] Sabala R.F., Usui M., Tamura Y., Abd-Elghany S.M., Sallam K.I., Elgazzar M.M. (2021). Prevalence of colistin-resistant *Escherichia coli* harbouring *mcr-1* in raw beef and ready-to-eat beef products in Egypt. Food Control.

[43] Hmede Z., Kassem I.I. (2018). The colistin resistance gene, *mcr-1*, is prevalent in commensal *E. coli* isolated from Lebanese pre-harvest poultry. Antimicrob. Agents Chemother..

[44] Hassan J., El-Gemayel L., Bashour I., Kassem I.I. (2020). On the edge of a precipice: The global emergence and dissemination of plasmid-borne *mcr* genes that confer resistance to colistin, a last-resort antibiotic. Antibiotics and Antimicrobial Resistance Genes in the Environment.

[45] Hmede Z., Alhaj Sulaiman A., Jaafar H., Kassem I.I. (2019). Emergence of plasmid-borne colistin resistance gene *mcr-1* in multidrug-resistant *Escherichia coli* isolated from irrigation water in Lebanon. Int. J. Antimicrob. Agents.

[46] Kassem I.I., Kehinde O., Kumar A., Rajashekara G. (2017). Antimicrobial-resistant *Campylobacter* in organically and conventionally raised layer chickens. Foodborne Pathog. Dis..

[47] Nisar M., Kassem I.I., Rajashekara G., Goyal S.M., Lauer D., Voss S., Nagaraja K.V. (2017). Genotypic relatedness and antimicrobial resistance of *Salmonella* Heidelberg isolated from chickens and turkeys in the midwestern United States. J. Vet. Diagn. Invest..

[48] Sahin O., Kassem I.I., Shen Z., Lin J., Rajashekara G., Zhang Q. (2015). *Campylobacter* in poultry: Ecology and potential interventions. Avian Dis..

[49] Cogliani C., Goossens H., Greko C. (2011). Restricting antimicrobial use in food animals: Lessons from Europe. Microbe.

[50] Kimera Z.I., Mshana S.E., Rweyemamu M.M., Mboera L.E., Matee M.I. (2020). Antimicrobial use and resistance in food-producing animals and the environment: An African perspective. Antimicrob. Resist. Infect. Control.

[51] Hassan J., Kassem I.I. (2020). Audacious hitchhikers: The role of travel and the international food trade in the global dissemination of mobile colistin-resistance (*mcr*) genes. Antibiotics.

